# Farmer Health and Adaptive Capacity in the Face of Climate Change and Variability. Part 2: Contexts, Personal Attributes and Behaviors

**DOI:** 10.3390/ijerph8104055

**Published:** 2011-10-21

**Authors:** Anthony Hogan, Adam Bode, Helen Berry

**Affiliations:** 1 School of Sociology, College of Arts and Social Sciences, The Australian National University, ACT 0200, Canberra, Australia; E-Mail: adam@ancd.org.au; 2 Centre for Research and Action in Public Health, The University of Canberra, University Drive, Bruce, ACT 2601, Australia; E-Mail: helen.berry@canberra.edu.au

**Keywords:** climate change, farmers, health, adaptation, social profiles

## Abstract

This study extends the emerging body of research on farmer adaptation to climate change, by segmenting farmers on the basis of specific attributes (health, values, belief about climate change, sense of responsibility for climate change, desire to change, social, human and financial capitals and farmer demographics) and considering such attributes as critical social aspects of the contextualized capacity to adapt. The segmental analysis was based on a nationally representative sample of 3,993 farmers concerned with farmer adaptation of climate risks. The resulting data were subjected to two-step cluster analysis to identify homogenous groups of farmers based on factors related to climate change adaptation. A three-cluster solution was identified wherein farmers were distinguishable on the basis of belief in climate change, desire for financial assistance and advice, social connectedness, information seeking, and adverse farm conditions. The largest group (Cluster 1: 55%) was characterized by farmers who recognized being affected by drought and drying and who were actively engaged in adaptive practices, despite the fact that they had little income and poor farm resources. One third of these farmers reported that their health was a barrier to sustained activity in farming. Cluster 2 (26%) was characterized by farmers not readily affected by drying, who enjoyed good incomes, good health and better farming conditions. They expressed little desire to adapt. The smallest cluster (Cluster 3: 19%) was also characterized by farmers who recognized that they were affected by drying. However, despite a desire to adapt, they had very little means to do so. They reported the poorest natural resources and the poorest health, despite being younger. The findings suggest that it is the intent to adapt, starting from where people are at, which is a more important indicator of the capacity to work towards sustainable practices than assets tests alone.

## 1. Introduction

It is well-understood that all farmers are not the same [1] and that systematic differences between types of farmer are of practical importance. As Emtage *et al.* [2] point out: “rural landholders vary considerably in their socio-economic characteristics, values and capacity” and an understanding of these factors is needed for successful engagement and development programs. Early and mostly qualitative approaches to understanding cultural and socio-economic differences among farmers and landholders [3,4] were concerned with structural and cultural attributes of adaptive decision-making. The structural aspect of this work [5–11] reflects on long standing sociological insights which observe that external (for example, land size and production systems [12]), environmental (for example, climate) and economic factors (including input costs and sales prices [9]) impinge upon farmers’ ability to make adaptive decisions. Research on the cultural aspects of decision making builds on the early work of van der Plog [13] which was concerned with theories of farming styles. This qualitative work (e.g., [9,12,14,15]) identified that farmers exhibited a number of “major styles” [4] which ranged from traditional through to the innovative [12]. *Traditional* farmers were often older and had “been reasonably successful ... (who saw) no need for change” while *Innovators* were seen to be at ‘the forefront of agricultural change’ [14]. These researchers also reported on farmers whose style was strongly influenced by financial “impediments” and whose farms “may no longer be viable or lacked the financial backing to expand” [14].

A body of social research concerned with how farmers are adapting in the face of climate change is also beginning to emerge in the literature. Flemming and colleagues [16] recently qualitatively identified four distinct dimensions of importance (money, earth, human responsibility and questioning) which influence how farmers respond to climate change. Widcorp [17] quantitatively examined, at a regional level, farmers’ technical knowledge of, and attitudes towards, climate change, climate variability and greenhouse gas emissions. A part of the study examined farmer typographies which were based on climate factors, structural characteristics, geographic factors and attitudinal statements. Geographic features, such as farming in the peri-urban environment, featured strongly in their typologies and was a differentiating factor in three of the four typologies which they identified (*autonomous* with attributes of being older, self-reliant and not particularly concerned about climate change; *speculative—*primarily focused on short-term farming practices; *ambitious,* having the largest farms, being younger than the average, having mixed views on climate change, showing a readiness to diversify into other enterprises, valuing new technology and planning ahead; and *prudent* being well-educated farmers ready to take on new ideas and technologies, but who were risk-averse). Marshall and Marshall [18] qualitatively identified four attributes of adaptive capacity among fishers (perception of risk associated with change; the ability to plan, learn and reorganize; proximity to thresholds of coping; and level of interest in change). Marshall [19] quantitatively examined these attributes in a regional study of graziers in northern Australia, noting correlations between the adaptive attributes and components of resource dependency (e.g., financial aspects, attachment to occupation and place) but did not extend this work to segmenting the samples.

These studies make a strong case for the need to understand sub-populations of farmers. With climate change threatening the wellbeing and success particularly of Australia’s most at-risk farmers [20], systematic attitudes and values towards climate change among distinct sub-populations of farmers need to be considered: such factors ‘are shaped by (farmers’) individual and group-based interests, concerns and aspirations’ [21]. The extent to which climate change is seen to pose a threat to viability, for example, may vary across farmers [22].

The existing body of research on the adaptive capacity of farmers in the face of climate change has several limitations. Notwithstanding the quality and importance of the work conducted and the insights derived, the majority of the work which has been undertaken is qualitative. While these studies can provide richness, depth and local colour, they do not tend to (i) produce insights that can be confidently generalized to other places or populations or (ii) provide quantifiable effects that can be compared by place or population. The small amount of quantitative work which has been undertaken has been regional in nature, had small sample sizes, has not used cutting-edge quantitative techniques or has not attempted to empirically derive cluster structures and consider their consistency with the findings of qualitative studies. Moreover, none of the studies considered the role farmer health may play in influencing farmers’ approaches to climate change. Thus there is now a substantial need for large quantitative studies which can address some of these limitations and provide an empirically, quantifiable and integrated assessment of the many factors noted above.

The present study therefore extends the emerging body of research on farmer adaptation to climate change by empirically segmenting farmers on the basis of specific attributes (health, values, belief about climate change, sense of responsibility for climate change, desire to change, farmer demographics, and social, human and financial capitals; see methods section) and considering such attributes as critical social aspects of the contextualized capacity to adapt. In brief, then, this study examines whether there are indeed different types of farmers and, if so, explores how they differ with respect to factors which may be associated with how they may act to address climate change.

## 2. Methods

This paper extends the analysis reported in Part 1 of this study [23] and examines the extent to which farmers may differ systematically on specific attributes (health, values and belief about climate change, and social responsibility, and the desire to change assessed within the context of specific capitals and farmer demographics) where such attributes may be considered as critical social aspects of the capacity to adapt.

### Data

Details of the data used in this study are presented in Berry *et al.* [23]. Briefly, participants were 3,993 Australian farmers who voluntarily completed a mailed questionnaire (for details, see [22,23]). The survey contained: (i) 115 items based on nine themes (condition of farm, on-farm adaptation, interest in alternative energy forms, attitudes to climate change, perceptions of climate impact, aspects of social capital, program participation, information usage and aspirations for future programs); (ii) 11 demographic items, such as farm size, type of agricultural production, irrigation status, age, sex, and on- and off-farm income; (iii) items tapping farm-related circumstances, including input costs and commodity prices, debt levels, interest rates, cash flow, farm income, labor, access to services and training, resource conditions and personal health as a barrier to adaptation; (iv) socio-demographic characteristics (sex, years of education post-Year 10, on-farm and off-farm income, self-assessed financial viability and age); and (v) farming-related variables (land size, irrigation status and main area of primary production). A further health item, ‘my health and fitness’, was included to measure health status at the time of data collection. The items were reduced to a more parsimonious set of twenty key concepts and these were further reduced to five overarching concepts (belief in climate change; desire for financial assistance; social connectedness; information-seeking; and adverse farm conditions). In both cases, exploratory factor analyses followed by one-factor congeneric modelling were used sequentially to explore and refine the concepts. These procedures, and our rationale for their deployment, are detailed in Berry *et al.* [23]. At the time the data were collected, many parts of Australia were experiencing their worst drought on record.

### Analytic approach

Cluster analysis was used to conduct this study. Cluster analysis refers to a group of exploratory statistical analyses that identify homogenous sub-groups of respondents within a larger heterogeneous sample. Cluster analyses are conceptually similar to analyses that involve grouping variables or respondents such as principle components analysis, multi-dimensional scaling, and latent class analysis. Cluster analyses can be used in the development of hypotheses concerning complex and multifaceted concepts [24] and to identify factors influencing such complex and multifaceted concepts (for a review, see Berry *et al.* [25]). It is for these reasons that cluster analysis was selected for the analysis. All analyses were conducted using SPSS Version 18. Accurately weighted composite scores, based on weightings derived from one-factor congeneric modelling, were calculated for the five overarching concepts (belief in climate change; desire for financial assistance and advice; social connectedness; information seeking; and adverse farm conditions) and were used in the cluster analysis as continuous variables. A two-step cluster analysis was used in the study because it can accommodate mixed continuous and categorical data and large datasets, as was the case in this study. Previous studies have described the technical and methodological issues surrounding two-step cluster analysis (for a review and exemplar study, see [25]). Log-likelihood distances were used to measure distance between clusters in this study. As reported elsewhere, and as was the case in this study, cluster solutions are evaluated on the substantive criteria of: (i) meaningfulness, (ii) scientific usefulness, and (iii) parsimony (the fewest number of clusters necessary to produce a meaningful and statistically acceptable solution). Different types of farmers were identified using two-step cluster analysis based on the five continuous measures described above. Statistical criteria supplemented the substantive criteria. The first is one-off jumps in the ratios of change calculated from the agglomeration schedules (see [25]). These were assessed using two agglomeration schedules: Schwarz’s Bayesian Criterion and Akaike’s Information Criterion. The second statistical criterion employed is statistically significant difference in mean scores (for continuous variables) or distributions (for categorical variables) between clusters.

Second, and to assist reporting and interpretation, the five factors included in the cluster analysis were divided into low, medium, and high tertiles and reported in the context of the socio-economic positions of the respondents. As Smit and Wandel [22] point out, analyses such as these need to take into account the extent to which individual efforts and resources to adapt ‘may be constrained or even nullified by social, economic and political forces that effectively shape local vulnerabilities’.

## 3. Results

### 3.1. Three-Cluster Solution

The cluster analysis returned a three cluster solution. Examination of the two ratios of change revealed a steep jump between three and four clusters. Table 1 presents the criterion statistics and ratios of change for each agglomeration schedule. A steep jump in the ratios of change occurs between three and four clusters, indicating that three clusters is a statistically appropriate solution.

### 3.2. Cluster Descriptions

Table 2 presents the mean scores by cluster for the twenty concepts from which the five overarching concepts used in the cluster analysis were derived (these were: (i) belief in climate change, (ii) desire for advice and assistance, (iii) social connectedness, (iv) information seeking and (v) adverse farm conditions). Cluster memberships were 55% (Cluster 1 and subsequently named *Cash poor long term adaptors*), 26% (Cluster 2 and subsequently named *Comfortable non-adaptors*) and 19% (Cluster 3 and subsequently named *Transitioners*).

Table 3 presents cluster centroids with respect to the five cluster analysis variables. For ease of interpretation (see methods section), these continuous scores have been recoded by tertile split into three categories: low, medium and high corresponding, respectively, to the bottom, middle and top tertiles.

#### 3.2.1. Cluster 1 (“Cash-Poor Long-Term Adaptors”)

##### Primary characteristics

Cluster 1 was the largest group, constituting 55% of respondents. Nearly three quarters reported either a high (42%) or medium (31%) level of belief in climate change. Almost two-thirds (60%) of Cluster 1 reported a strong desire for financial help from government, with only 6% reporting low levels of desire for such assistance. More than half of Cluster 1 reported high levels of social connectedness (52%), with most of the remainder reporting medium levels of connectedness (37%). Cluster 1 was not inclined to use information sources, with more than half (57%) being lower level users. Cluster 1 farm conditions tended to be poor, with 49% reporting high levels of adverse farm conditions while only 15% reported low levels of adverse farm conditions.

##### Secondary characteristics

Cluster 1 had a mean age of 54 years. Three quarters (75%) reported that their health was good and 85% reported that they could cope with more change. Fifteen percent were women. Cluster 1 reported having large farms with an average size of 5,137 hectares. Nearly two-thirds (62%) of cluster 1 reported that the primary purpose of their farms was for business, as opposed to lifestyle purposes. A large proportion (79%) of Cluster 1 were involved in livestock production and 44% were involved in dry land cropping. Some (17%) were also involved in irrigated crops. Cluster 1 were substantially drought affected (83%). Almost half (45%) of Cluster 1 reported debt and market pressures impacting on their financial viability. Approximately one-third (30%) of Cluster 1 reported high or very high levels of cumulative on-farm risk, while one-quarter (26%) reported difficulties accessing farm support services. Nearly 30% reported that their health or fitness was a barrier to continuing to run their farms. Almost one third (30%) were considering the possibility of exiting the industry while one-quarter (24%) reported presently considering selling off or leasing out part of their property.

More than half of Cluster 1 (60%) regarded themselves as ‘breaking even’ or being financially viable based on the last five years. One-third (33%) of Cluster 1 reported on-farm income exceeding $40,000 while only one quarter reported off-farm income of more than $40,000 per annum. A large majority of Cluster 1 reported being under financial pressure in the face of adaptations demanded by climate change; only one quarter (23%) reported having had sufficient funds to make adaptations which they considered necessary. A substantial number of Cluster 1 members received government support. More than one-third (36%) received the Government’s Exceptional Circumstances (EC) interest rate subsidies, an additional one-third (35%) received EC drought relief payments, one-quarter utilized the EC advice and planning program and a small portion (12%) received irrigation management grants. Sixteen per cent of Cluster 1 had used the Rural Financial Counselling Service. Cluster 1 reported being well-connected people who sought out information and support. They were strong users of both on-line (62%) and non-electronic (58%) information sources.

#### 3.2.2. Cluster 2 “Comfortable Non-Adaptors”

##### Primary characteristics

Cluster 2 was the second largest group identified in the cluster analysis, making up just over one-quarter (26%) of respondents. They tended towards not believing in climate change, with two-in-five (39%) reporting low levels of belief. A large majority (84%) of Cluster 2 reported a low desire for financial help from government with less than 1% reporting high levels of desire for such assistance. Cluster 2 showed evidence of strong social connectedness with nearly one-half (45%) reporting high levels of connectedness and one-third (34%) reporting medium levels of connectedness. Cluster 2 were strong users of on-line information sources (84%). Cluster 2 farm conditions also tended to be very good, with more than three-quarters (79%) falling in the low group for adverse farm conditions. Only 3% were in the top group in which farm conditions were poor.

##### Secondary characteristics

Cluster 2 reported having farms with an average lot size of 1,639 hectares and reported a mixed response with regards the primary purpose of their farm: 40% reported that the purpose of their farm was equally for business and lifestyle with a small majority (56%) reporting lifestyle as an important component of operating their farm. More than three-quarters of this group (77%) were involved in livestock production and one-quarter in dry land cropping. Sixteen per cent identified themselves as irrigators. At the time of data collection, more than one-half (55%) of Cluster 2 were in drought. Cluster 2 were an older group of farmers with two-in-five (39%) aged 64 years or older. A small proportion (13%) of Cluster 2 were female. Three-quarters of Cluster 2 (74%) reported that their health was good and nearly all (87%) reported that they could cope with more change.

With the exception of facing market pressure (92%), members of this group did not report problems with on-farm conditions. One-in-ten (9%) reported substantial debt pressures or difficulties accessing support services. In terms of cumulative on-farm risk factors (e.g., poor soil, low water, high debt levels, labor problems), these farmers reported a low number of total risk problems; only 3% reported high or very high levels of on-farm risks. As a group, Cluster 2 was not concerned about their financial viability in the face of climate change. Seventeen per cent reported that their health or fitness was a barrier to continuing to run the farm. Few members of this group (7%) reported difficulties accessing farm support services and only 6% used the Rural Financial Counselling Service. Similarly, Cluster 2s usage of government assistance programs was low, with 6% utilizing interest rate subsidies, 9% utilizing EC relief payments and 5% utilizing EC assistance and related planning services. More of these farmers had taken up irrigation management grants, but they were still in the minority (45%). A majority (56%) reported themselves as breaking even or being financially viable based on the last five years. One-third (32%) of these farmers reported on-farm incomes of over $40,000 per annum and 35% reported off-farm income over $40,000 per annum.

Across the range of possible planned approaches to risk management, three-quarters (75%) of Cluster 2 consistently reported that they were only considering undertaking risk management activities with 11% doing so only as part of a short-term response. Although one-half of this group (51%) reported having had sufficient funds to make adaptations which they considered necessary, few were interested in making climate change-related adaptations. They reported that they were not very interested (71%) in producing green power. Cluster 2 was also not interested in learning about sustainable practices (67%). While a large minority (25%) of this group was considering the possibility of leaving the industry, only a small proportion (14%) of Cluster 2 reported considering selling off or leasing out part of their property.

Cluster 2 reported being socially well-connected and were high users of both on-line (84%) and non-electronic information sources (93%). They tended to agree that there was a moral responsibility in the community to reduce GHEs (77%). As a group, they leaned towards agreeing that climate change existed (56%), although they did not readily acknowledge physical changes in the environment (such as more frequent extreme weather events) as evidence of climate change (64%).

#### 3.2.3. Cluster 3 (“Transitioners”)

##### Primary characteristics

Cluster 3 made up the smallest cluster grouping representing 19% of respondents. Most members of Cluster 3 believed in climate change with nearly two-in-five (39%) scoring in the ‘high belief’ group on this factor. Members of Cluster 3 were mixed in their desire for financial help from government. While approximately one-quarter (26%) reported low levels of desire for such assistance, another one-third (36%) reported high levels in desire for assistance. Cluster 3 reported the lowest levels of social connectedness with almost all (96%) respondents falling in the low grouping. Cluster 3 were also less inclined to use information sources, with more than one-half (57%) reporting being low level users. Cluster 3 farm conditions also tended to be poor, with two-thirds (65%) falling in the group with high levels of adverse farm conditions.

##### Secondary characteristics

Cluster 3 reported a mean lot size of 2,746 hectares with a majority (59%) reporting that the purpose of their farm was business. Of these farmers, three-quarters (74%) reported being involved in livestock production and one-third (35%) in dry land cropping. Around one-sixth (17%) were also involved in irrigated crops, with small minorities in dairy (9%) and intensive farming (4%). At the time of the survey, the large majority (82%) of Cluster 3 were in drought. Almost one-half of these farmers (44%) reported high to very high levels of cumulative on-farm risk and one-third (35%) reported difficulties accessing farm support services. More than one-half of this group (52%) was aged under 55 years and one-fifth (22%) was aged 45 years or younger; 17% were women farmers. Cluster 3 reported poor health, with fewer than one-half (47%) reporting that their health was good; one-in-three (35%) reported that their health or fitness was a problem in being able to continue to operate the farm. A significant minority of Cluster 3 (19%) agreed that they ‘could not cope with any more change’, with only a minority positively agreeing that they could.

Almost one-half (49%) of these farmers regarded themselves as breaking even or being financially viable based on the last five years. These farmers reported the lowest levels (27%) of on-farm income exceeding $40,000 per annum and a minority (28%) reported off-farm income of more than $40,000 per annum. Almost one-half the farmers in this group (46%) reported debt pressures and market pressures impacting on their viability. Only 10% of this group reported utilizing the rural financial counselling service. Nearly one-quarter (23%) of Cluster 3 received an interest rate subsidy, one-quarter (25%) received EC payments and around one-in-ten had received EC advice and planning (12%) and irrigation management grants (9%). A small proportion (13%) of Cluster 3 reported having sufficient funds to make adaptations which they considered necessary, while a large proportion (41%) were considering leaving the industry. Similarly, almost one-third (31%) of Cluster 3 reported considering selling or leasing out part of their property.

As a group, Cluster 3 showed interest (56%) in initiatives that would help make them sustainable in the long term and two-thirds (67%) were interested in incentives to take up more fuel-efficient machinery. However, approximately one-half of Cluster 3 did not use the internet (47%) or consult industry groups (52%) about their on-farm decisions. Only one-quarter utilized the rural farm press and one-third consulted friends and other farmers when making these decisions. Cluster 3 agreed that there was a moral responsibility to reduce GHEs and acknowledged changes in the physical environment (such as more frequent extreme weather events) as evidence of climate change. However, their attitudes to climate change were varied: more than one-half (53%) believed that climate change was real, while only (21%) did not; the remainder were uncertain.

## 4. Limitations of this Study

Though the study was based on one of the largest (and most representative) reported surveys on farmers conducted to date, we urge caution with respect to certain limitations. First, the variables used in the study were not derived from previously empirically validated measures or scales. However, the use of multiple exploratory and confirmatory factor analytic techniques generated scientifically valid, meaningful and useful variables with which to investigate the research question, and which contributed to an innovative solution that extends knowledge and has policy applicability. Second, though we had available sociodemographic data, more detailed water and environmental information would have been helpful in characterizing cluster members. Finally, we did not have geocoding for our participants and were not, therefore, able to integrate the unique characteristics of Australia’s geography, climate, agricultural sector, and policy-responses to climate change.

## 5. Discussion and Conclusions

This study makes a number of important contributions to the existing literature. First, the study identified three distinct subtypes of farmers in a nationally representative sample of Australian farmers. As an aside, we note that this study provides support for the view that ‘systematic variation between types of farmers’ [20] may be masked by the reporting of averaged survey scores and that future research should segment respondents and more closely examine the conditions of those who may be at risk. In doing this, we found that the human aspects of adaptive capacity, based on the capacity to cope with change, farmer health, social connectedness, and the ability and readiness to use information, uniquely and powerfully differentiated types of farmers from one another. We note that similarities are evident between the sub-populations of farmers identified in this study and sub-populations reported in other studies that we have noted in this paper. Such cross-study commonalities include recognition of older and better-resourced farmers who take a more traditional approach to farming, as well as the presence of innovators and adaptors. Structural barriers, such as the condition of the natural assets and access to financial resources, were also evident across studies, as were sub-populations in which cluster members were aware of climate risks. Of importance, but also of particular concern, was the fact that specific sub-populations who are particularly vulnerable to climate change and other stressors were identified in these studies. Our findings are consistent with and, particularly by including a health perspective, add value to these preceding studies.

Second, to develop this theme further, our findings provide clear empirical support for the perspective that farmers are not an homogeneous group [1], that one-size-fits-all assessments of farmer vulnerability are inappropriate [26], that the adaptive capacity of individuals cannot be isolated from broader socioeconomic and environmental determinants [22], and that a variety of distinctive attributes are evident among farmers [16,17,19,27], attributes which will vary depending on the analytical approach taken to the study.

Third, the findings of this study provide empirical support for the view that scientific attempts to understand farmer readiness to adapt need to go well beyond a narrow view of farmer assets: along with financial capital, vital concepts, such as connectedness, health and education, must be considered not just desirable but essential farmer assets [26]. Of particular note, it must be clearly understood that farmer health is a powerful contributor to adaptive capacity and intention, with poor health presenting a formidable barrier to both. Yet we also found, movingly, that health is not a barrier to *wanting* to adapt and that many poorly-resourced and less-than-well farmers could perceive the need to adapt and to make whatever changes they could manage. This suggests that providing, where needed, targeted health-related support for farmers could represent an effective climate change adaptation intervention—not to mention its potential to offer an innovative approach to health policy. Meanwhile, farmers with the most resources, best health and highest levels of capitals showed the *least* interest in adapting to climate change. With climate science indicating that climate change will bring continuing (mainly adverse) change to climate patterns in Australia, their lack of interest in adaptation may, over time, evolve [28].

The sub-populations of farmers described in this study made it evident that farmers’ assessments of their situation were influenced by the material and social circumstances in which they found themselves, by their health and by the extent to which they saw themselves as being affected [29] by climate change. It was also notable that the group facing (and not shirking) the greatest challenges to adaptation were working on the poorest farms and had the poorest health, and that those with the least inclination towards adaptation enjoyed good farming conditions and better health. Similar observations can be made with regard to income, poverty, access to services and information, and social connectedness. Notably, and we reiterate this pivotal point, those who emerged as being the worst affected by drought and drying were also those with the greatest desire, *irrespective of their financial, health and other assets*, to engage with change. Perhaps it is the intent to adapt, starting from where people are at, which is a more important indicator of the capacity to work towards sustainable practices [22,30]; it behoves governments and service providers to honour this intent, particularly when it is made in the face of considerable adversity.

## Figures and Tables

**Table 1:** Criterion statistics and ratios of change for each agglomeration schedule.

Number of Clusters	Schwarz’s Bayesian Criterion (BIC)	BIC Change a	Ratio of BIC Changes b	Ratio of Distance Measures c
1	13919.106			
2	11998.433	−1920.673	1.000	1.668
3	10880.499	−1117.934	0.582	1.640
4	10230.971	−649.528	0.338	1.235
5	9720.952	−510.019	0.266	1.062
6	9245.639	−475.314	0.247	1.401
7	8930.234	−315.405	0.164	1.303
8	8707.359	−222.875	0.116	1.004
9	8485.603	−221.755	0.115	1.111
10	8294.409	−191.195	0.100	1.214
11	8151.516	−142.893	0.074	1.097
12	8028.680	−122.835	0.064	1.277
13	7950.458	−78.222	0.041	1.104
14	7887.425	−63.033	0.033	1.031
15	7828.816	−58.609	0.031	1.068

a The changes are from the previous number of clusters in the table

b The ratios of changes are relative to the change for the two cluster solution

c The ratios of distance measures are based on the current number of clusters against the previous number of clusters.

*Note*. Line in table represents cut-point.

**Table 2:** Mean scores on 20 key concepts by cluster group membership.

Latent concept	Comfortable non-adaptors	Cash poor longer term adaptors	Transitioners	Std. error
1. Barriers accessing support services	2.63	2.41	2.68	0.02
2. Debt pressures	2.19	3.54	3.53	0.02
3. Conditions of on farm resources	1.88	2.62	2.59	0.02
4. Market pressures on viability	4.19	4.74	4.74	0.01
5. Risk management	2.35	3.15	2.63	0.02
6. Withdrawing from the industry	1.82	1.92	2.09	0.02
7. Intention to adapt practices	2.55	3.19	2.77	0.02
8. Desire to produce green power	1.97	2.33	2.16	0.02
9. Moral responsibility to reduce Greenhouse Gas Emissions (GHEs)	3.87	4.04	3.79	0.02
10. Believe climate change (CC) is real	3.39	3.43	3.51	0.02
11. Concerned about financial viability in the face of CC	2.95	3.91	3.95	0.02
12. Notice evidence of CC	3.15	3.35	3.47	0.02
13. Confident about coping	3.89	3.77	3.24	0.01
14. Trust	2.66	2.62	2.56	0.01
15. Financial help and advice	4.78	3.80	4.23	0.02
16. Advice from rural organizations	4.10	3.39	4.16	0.02
17. Help make my farming practices more sustainable	2.94	4.12	3.65	0.02
18. Offering direct financial assistance	2.54	4.46	4.03	0.02
19. Non-electronic information source	3.79	4.19	3.16	0.01
20. Online information sources	3.66	4.13	3.63	0.01

Note. All factors significantly differ at p > 0.001. For items 1–5 higher scores reflect greater problems. For the remaining items higher scores indicate greater agreement with the statements presented. All items were scored on 1–5 likert scales except for item 5, which were scored on 1–4 (for further details see Hogan *et al.* 2010).

**Table 3:** Comparison of clusters by key cluster variables.

Indicator	Comfortable non-adaptors (%)			Cash poor longer term adaptors (%)			Transitioners (%)			χ^2(4)^	*P*-value
			
	L	M	H	L	M	H	L	M	H		
Belief in climate change	38.6	27.2	34.1	26.9	31.0	42.1	28.6	32.1	39.2	48.4	*P* < 0.001
Desire for government financial help	84.3	15.1	0.6	5.5	34.3	60.3	26.2	38.0	35.8	2200.1	*P* < 0.001
Social connectedness	21.7	33.9	44.5	10.5	37.4	52.1	95.9	41.1	0.0	2038.4	*P* < 0.001
Use of information sources	46.6	29.0	24.4	56.7	29.8	13.5	56.7	29.8	13.5	855.4	*P* < 0.001
Adverse farm conditions	78.8	18.1	3.1	14.7	36.7	48.6	7.6	27.1	65.3	1728.3	*P* < 0.001
